# Does intra-ruminal nitrogen recycling waste valuable resources? A review of major players and their manipulation

**DOI:** 10.1186/s40104-018-0249-x

**Published:** 2018-04-22

**Authors:** Thomas Hartinger, Nina Gresner, Karl-Heinz Südekum

**Affiliations:** Institute of Animal Science, University of Bonn, 53115 Bonn, Germany

**Keywords:** Ammonia, Bacteria, Deamination, Hyper-ammonia producing, Nitrogen efficiency, Protein, Proteolysis, Protozoa, Ruminant

## Abstract

Nitrogenous emissions from ruminant livestock production are of increasing public concern and, together with methane, contribute to environmental pollution. The main cause of nitrogen-(N)-containing emissions is the inadequate provision of N to ruminants, leading to an excess of ammonia in the rumen, which is subsequently excreted. Depending on the size and molecular structure, various bacterial, protozoal and fungal species are involved in the ruminal breakdown of nitrogenous compounds (NC). Decelerating ruminal NC degradation by controlling the abundance and activity of proteolytic and deaminating microorganisms, but without reducing cellulolytic processes, is a promising strategy to decrease N emissions along with increasing N utilization by ruminants. Different dietary options, including among others the treatment of feedstuffs with heat or the application of diverse feed additives, as well as vaccination against rumen microorganisms or their enzymes have been evaluated. Thereby, reduced productions of microbial metabolites, e.g. ammonia, and increased microbial N flows give evidence for an improved N retention. However, linkage between these findings and alterations in the rumen microbiota composition, particularly NC-degrading microbes, remains sparse and contradictory findings confound the exact evaluation of these manipulating strategies, thus emphasizing the need for comprehensive research. The demand for increased sustainability in ruminant livestock production requests to apply attention to microbial N utilization efficiency and this will require a better understanding of underlying metabolic processes as well as composition and interactions of ruminal NC-degrading microorganisms.

## Background

Understanding the rumen metabolism is of central importance [1, 2] and a prerequisite to meet the animal’s requirements for nutrients and energy. The rumen microbiota constitutes a complex ecosystem, the metabolic activity of which is responsible for rumen metabolism, including intra-ruminal N recycling [3]. Thus, it is a key factor that needs to be taken into consideration when a sustainable and efficient livestock production is pursued. Rumen microbiota-related studies have so far focused on cellulolytic microorganisms, their metabolic pathways and how to optimize ruminal fiber degradation [4, 5]. However, in the rumen, the vast majority of dietary crude protein is microbially degraded to ammonia [6], which constitutes the main and sometimes even sole N source for rumen microorganisms [7]. Excessive ruminal proteolysis and deamination cause inordinate amounts of ammonia, which are absorbed by the ruminant, converted to urea and subsequently predominantly excreted via the urine, leading to increased environmental pollution [8] and poor amino acid (AA) supply to the host. Hence, an efficient utilization of crude protein should be aimed at; also, to ensure the maximum retention of N, knowledge of ruminal NC-degrading microorganisms is indispensable [9].

Research on the abundance, composition and metabolism of NC-degrading microorganisms is particularly needed for developing strategies to cope with the challenge of finding the optimal balance between the inhibition of ruminal NC degradation, without compromising post-ruminal AA absorption, and the simultaneous provision of appropriate amounts of N for the rumen microbiota. Improvements in techniques for studying microbial communities already allow the broad use of culture-independent techniques [10], which enable a more comprehensive characterization of the rumen microbiota compared to cultivation [11]. Omics-based approaches and quantitative real-time polymerase chain reaction (qPCR) assays have markedly enhanced our understanding of the rumen microbiota and are inevitable for the investigation of NC-degrading microorganisms. In particular, when omics methods are combined to analyze not only the abundance and diversity of genes, but also functional compositions as well as protein and metabolite profiles, a deeper knowledge will be obtained [10]. However, as omics approaches are not sufficient to target microorganisms on a species level [12] or to determine absolute abundances [13], qPCR represents an indispensable tool for the investigation of single microbial key species of ruminal NC degradation [8, 14].

The present review represents a starting point and aims to encourage research targeting the lack of knowledge of NC-degrading microorganisms, thereby developing and optimizing strategies for manipulating them. To give a critical status quo on this topic, existing information on the activity and abundance of rumen microorganisms involved in the degradation of proteins, peptides, AA and urea, as well as their principal interactions, is briefly summarized in the first part of this review. So far, this information is limited and needs expansion by state-of-the-art technologies regarding all aspects of the rumen microbiome, i.e. genome, transcriptome, proteome and metabolome, finally leading to a better understanding of both, its structure and its function. Thereby, functional characterization by omics addressing also uncultivable microorganisms will expand our current knowledge on NC-degrading microbes that was generated predominantly by cultivation and enzymatic activity tests. The second review part will cover options that have been considered so far to influence NC-degrading microorganisms by dietary factors as well as vaccination. Here, we based our review on a systematic literature search, as the high diversity in experimental conditions and applied techniques between the contemplated studies make a meta-analysis inappropriate.

## Ruminal microorganisms involved in the degradation of nitrogenous compounds

**Fig. 1:**
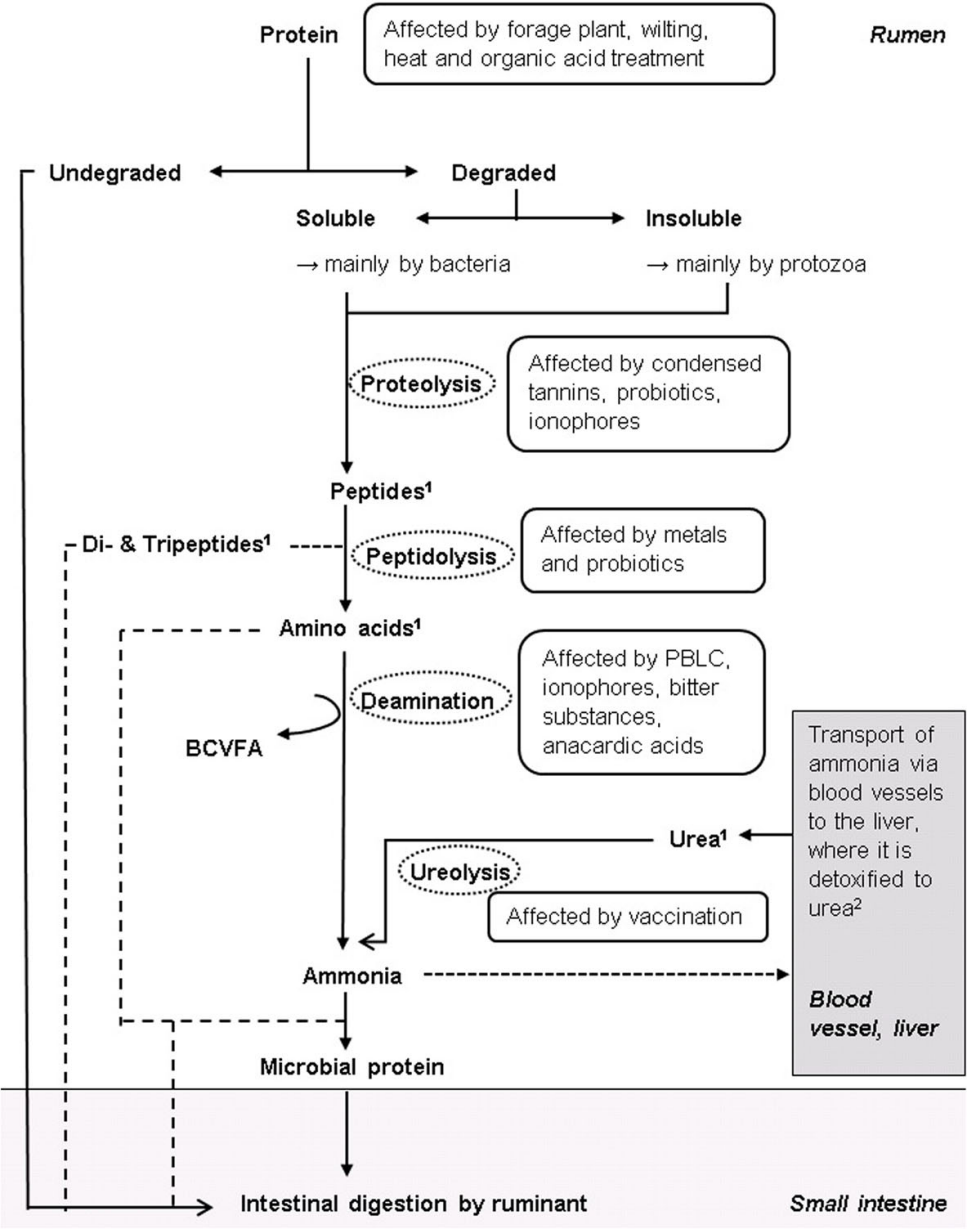
Simplified scheme of intestinal N metabolism and the target sites of manipulation strategies for reducing ruminal NC degradation that have shown effectiveness in vivo or in vitro (according to [6, 22, 86, 87, 102, 138, 151, 155, 156, 160, 166, 168, 202, 208, 218]). ^1^This NC can also be supplied with the feed; ^2^Urea is partly excreted with urine

Rumen microbes are supplied with NC by the diet and with that mainly as proteins, peptides and AA. In addition to the potential provision of urea by feed [6, 15], endogenous urea is supplied to the rumen via the rumino-hepatic circulation [16]. Depending on NC, different ruminal microorganisms are involved in their breakdown (Table 1) and synergistic microbial enzyme activities are often required for the complete degradation of NC to ammonia [17, 18] (Fig. 1). However, one has to emphasize that published studies quantifying the abundances of microorganisms are very heterogenic in their sampling, as well as quantification methods, thus complicating their comparison. High standardization of experimental conditions help to diminish this problem and should be considered in future study designs. As bacterial, protozoal and fungal cells contain different copy numbers of 16S rDNA, 18S rDNA or internal transcribed spacer 1, respectively [19–21], it is particularly difficult to put data from culture-independent approaches in relation to earlier results obtained from cultivation. Moreover, culture-independent techniques allow species-specific identification, but rumen microorganisms are often only characterized on a genus level, e.g. *Prevotella* by Deckardt et al. [22]. Due to the great heterogeneity within one genus [23], the interpretation of such results becomes even more challenging and a considerable part of the potentially acquired information is easily lost.

**Table 1:** Overview of microorganisms involved in the ruminal degradation of proteins, peptides, AA and urea^a^

Group	Microbial species	Proteins	Peptides	AA	Urea	Reference
Bacteria	*Allisonella histaminiformans*			X		[72, 73]
	*Butyrivibrio fibrisolvens*	X		X		[26, 69]
	*Butyrivibrio proteoclasticus*	X				[27]
	*Clostridium* sp*.*			X		[77]
	*Clostridium aminophilum*			X		[77]
	*Clostridium sticklandii*			X		[77]
	*Eubacterium* sp.	X		X		[47, 69]
	*Eubacterium budayi*	X				[27]
	*Eubacterium pyruvativorans*			X		[82]
	*Eubacterium ruminantium*		X			[53]
	*Fibrobacter succinogenes*		X			[53]
	*Fusobacterium* sp.	X				[26]
	*Howardella ureilytica*				X	[91]
	*Klebsiella aerogenes*				X	[88]
	*Lachnospira multipara*	X	X			[49, 53]
	*Lactobacillus casei* var. *casei*				X	[88]
	*Micrococcus varians*				X	[89]
	*Megasphaera elsdenii*		X	X		[53]
	*Peptostreptococcus anaerobius*			X		[77]
	*Prevotella* sp.	X	X	X		[54, 70]
	*Prevotella albensis*	X	X			[39, 61]
	*Prevotella brevis*	X	X			[52]
	*Prevotella bryantii*	X	X			[52]
	*Prevotella ruminicola*	X	X	X		[51, 70]
	*Ruminobacter amylophilus*	X				[28]
	*Selenomonas ruminantium*	X		X	X	[15, 42, 69]
	*Staphylococcus* sp*.*				X	[88]
	*Staphylococcus saprophyticus*	X		X	X	[89]
	*Streptococcus bovis*	X	X	X		[27, 53, 69]
	*Streptococcus faecium*				X	[88]
Protozoa	*Dasytricha* sp.	X				[96]
	*Dasytricha ruminantium*	X	X			[94, 104]
	*Entodinium* spp.	X	X			[103, 104]
	*Entodinium caudatum*	X		X		[103]
	*Entodinium simplex*	X				[96, 103]
	*Epidinium* sp.	X				[96]
	*Epidinium caudatum ecaudatum*	X				[94, 96, 104]
	*Isotricha* spp.	X	X			[94, 96]
	*Ophryoscolex caudatus*	X				[94]
	*Polyplastron multivesiculatum*	X				[94]
Fungi	*Neocallimastix frontalis*	X	X			[114, 118]
	*Neocallimastix patriciarum*	X				[116]
	*Orpinomyces joyonii*	X				[116]
	*Piromyces* sp.	X	X			[118]
	*Piromyces communis*	X				[116]

^a^Without consideration of detection method, quantity of substrate degradation or impact on ruminal N metabolism

## Bacteria

### Proteolytic bacteria

Bacteria represent the most abundant domain in the rumen [24] and are present in numbers of 10^10^–10^12^ cells/g rumen content [2, 11]. In cattle, using rRNA-targeted oligonucleotide probes, Lin et al. [25] assigned 60–84% of total rRNA to this domain, while Prins et al. [18] had previously assigned 65% of ruminal proteolytic activity to bacteria, which underlines their involvement in NC degradation.

Two important key species for protein degradation belong to the highly abundant genus *Butyrivibrio* [24], namely *Butyrivibrio fibrisolvens* [26] and *B. proteoclasticus* [27]. They are present in ruminant species across all continents [24] and exert high proteolytic activities [27–29]. The increased number of 16S rDNA copies of *B. fibrisolvens* when protein supply to dairy cows was increased [30] may confirm its role in protein metabolism, and in sheep *B. fibrisolvens and B*. *proteoclasticus* accounted for approximately 4.2% and 4.0% of total 16S rDNA copies, respectively [31]. Besides proteolysis, *B*. *fibrisolvens* is also involved in fiber degradation [32]. The results of Vasta et al. [31] regarding the abundance of *B*. *proteoclasticus* are in accordance with qPCR data from Paillard et al. [14], whereas Reilly et al. [33] observed *B*. *proteoclasticus* to represent 2.01 × 10^6^/mL to 3.12 × 10^7^/mL, which corresponds to only 0.3% of the bacterial population [14]. This could be due to differences in fed diets [24]; however, the application of different primers or DNA extraction procedures can also cause diverse results [34, 35]. In this context, “a universal extraction method with equally efficient lysis of cell walls of all possible microorganisms” [36] is essential to obtain comparable results and calls for mandatory bead beating, particularly as the rumen harbors various hard-to-lyse bacteria [37].

*Streptococcus bovis* expressed extracellular proteases [26, 27, 29, 38] and high proteolytic activity in the presence of several proteins [38, 39]. According to Attwood et al. [27], *Strep. bovis* may be particularly significant for ruminal proteolysis in grazing ruminants due to the semi-continuous grazing pattern and high protein contents of pasture, which would provide unique conditions, enabling this species to become a dominate proteolytic bacterium. Nevertheless, *Strep. bovis* can be absent from the rumen [40] or account for only 0.5–1.6% of the ruminal bacterial DNA [30]. However, low abundant microorganisms can also exert high enzymatic activities [41] and are therefore essential for ruminal protein metabolism. Besides protein degradation, *Strep*. *bovis* degrades starch for glucose fermentation and exerted proteolytic activity independent of the available N source, which led to the hypothesis that *Strep*. *bovis* degrades protein not only for subsequent N utilization, but mainly to break down protein matrices, surrounding starch granules [38].

Other bacteria involved in ruminal protein degradation are *Selenomonas ruminantium* [42] and *Ruminobacter amylophilus* [28], although both show low abundance when quantified via quantitative fluorescence in situ hybridization in cattle [43] or qPCR in sheep [44] and cattle [45]. However, despite its low abundance, *Rb*. *amylophilus* is assumed to be a highly proteolytic bacterium of the rumen microbiota [46] and showed higher azocasein degradation rates than some *B. fibrisolvens* strains [28]. Species of *Eubacterium*, especially *Eubacterium budayi* [27], are further active protein degraders [47] and *Eubacterium* contributed 16% to total proteolytic activity in the rumen [27]. Analyzing ruminal bacteria by competitive PCR in dairy cows, Reilly et al. [47] found that approximately 0.3–0.9% of bacterial cells belonged to *Eubacterium*. Also, Fusobacteria have high proteolytic activities [26], but their contribution to ruminal protein degradation in high-yielding dairy cattle may be limited as next-generation sequencing (NGS) obtained no *Fusobacterium* sp. in heifers fed high-grain diets [48].

Several species of *Prevotella* are crucial for hydrolyzing dietary protein in the rumen [49]. For example, *P*. *albensis* exerted proteolytic activity when incubated with varying concentrations of casein [39]. Thereby, it had a lower specific proteolytic activity than *Strep. bovis,* but as *Prevotella* is highly abundant in the rumen [24, 50], the contribution of *P*. *albensis* to ruminal proteolysis is substantial [39]. Further studies showed that strains of *P*. *ruminicola* [51], *P. brevis* and, to a smaller extent also *P*. *bryantii,* possessed proteolytic activities [28, 52]. However, *Prevotella* is even more important in the subsequent degradation of peptides [53, 54] and will therefore be considered again in the following section.

Finally, there are various rumen bacteria with minor proteolytic activities [26, 42], e.g. *Lachnospira multipara* [49]. However, these microbes may be relevant for overall proteolytic capacity in the rumen, especially as they can have nutritional interdependences with highly proteolytic species [42].

### Peptidolytic bacteria

Peptides originating from the diet or ruminal proteolysis are mainly degraded by members of the rumen microbiota. As found for protease activity [18], peptidase activity is also predominantly of bacterial origin [54].

*Prevotella* represents a highly abundant genus in the rumen [24, 55] and was observed to have a broad peptidolytic activity [53, 54] with high peptidase diversity as recently obtained from metagenomic sequence data [56]. Stevenson et al. [57] observed that *Prevotella* spp. were highly abundant in the rumen of lactating cows and accounted for 42–60% of total eubacterial rDNA copies. Although *Prevotella* primers from [57] were later found to match numerous non-*Prevotella* species [58], the results are in the same range observed by NGS, i.e. 52% of all reads [55]. On a species level, van Gylswyk [49] stated that *P. ruminicola* accounted for up to 60% of total rumen flora when cultivating bacteria from rumen ingesta on several media. Although *P*. *ruminicola* is a predominant microbe in the rumen [59], this abundance should be overestimated, as cultivation can produce biased results, e.g. due to cells that are in a viable but non-culturable condition [60]. Culture-independent approaches quantified the classical members *P*. *bryantii*, *P*. *brevis* and *P*. *ruminicola* to be 2–5% relative sequence abundance [57]. The exceptionally huge deviation in the abundance of *P. ruminicola* between these studies must be considered critically as it barely represents normal variation between different rumen microbiota. Therefore, the ruminal abundance of specific *Prevotella* species demands further elucidation.

By expressing several peptidases with different substrate specificities [54, 61], *P*. *albensis* constitutes a central peptidolytic species in the rumen and pure cultures of *P*. *albensis* and *P. bryantii* expressed peptidase activities higher than or similar to those of rumen fluid when incubated with several peptides [53, 62]. In addition, several *Prevotella* species are potent carbohydrate degraders harboring a variety of CAZymes [63].

Although *Megasphaera elsdenii* lacks peptidase activity [64], this species expresses high dipeptidase activity [53]. Thus, *M*. *elsdenii* is substantial for the sufficient breakdown of dipeptides to AA in the rumen, especially in the case of protozoa being absent as they suppress the growth of *M. elsdenii* [53]. Concerning the abundance of *M. elsdenii*, there is a high inter-individual variation between ruminants [50], with *M. elsdenii* not being detected by qPCR in steer rumen samples in some studies [40, 65]. However, just as many qPCR-based studies identified this species in the rumen of steers [66], dairy cattle [30, 67] and in vitro systems inoculated with bovine rumen fluid [8, 22, 68]. *Ruminobacter amylophilus* and *Strep*. *bovis,* as well as *Lachnospira multipara, Fibrobacter succinogenes* and *Eu. ruminantium,* express weak peptidolytic activities [53], but their contribution to ruminal peptidolysis appears marginal.

### Deaminating bacteria

Only a small amount of AA is directly utilized for microbial protein synthesis. The bulk is deaminated, with volatile fatty acids, ammonia, carbon dioxide and methane being the end-products [6]. Total ruminal deamination is the result of a broad microbial activity, as no microorganism degrades all AA, but each prefers certain ones [69].

In 1961, Bladen et al. [70] stated that predominant bacteria with low ammonia production rates were the main ammonia producers in the rumen. However, as several studies have observed low abundant bacteria with high deaminating capacity [71], it is assumed that ruminal AA deamination is performed by two bacterial fractions: the first one constitutes bacteria present in a high number with low or moderate deaminating activity of about 10–20 nmol ammonia/min/mg protein [41]. This fraction includes *Butyrivibrio fibrisolvens* and *P. ruminicola*, which underlines their central role in ruminal N metabolism, as well as *M. elsdenii* [64, 70]. Although strains of *M. elsdenii* vary significantly in their deamination capacity, some possess ammonia production rates which are comparable to the hyper-ammonia producing bacteria (HAB) [64], belonging to the second fraction of deaminating bacteria in the rumen. Also, *Allisonella histaminiformans* has the ability to decarboxylate histidine, which results in the formation of histamine and small amounts of ammonia [72]. However, it is questionable whether *Allisonella histaminiformans* is part of the native ruminal microbiota as results on its presence are inconsistent [72–75].

The second fraction of deaminating rumen microorganisms are bacteria present in a low number which exert high deaminating activities of more than 300 nmol ammonia/min/mg protein [41]. These microbes are designated as HAB. Due to their high deaminating rates, they are of particular relevance for intra-ruminal N recycling [71]. Although ammonia is an essential N source for cellulolytic rumen microbes [7], an oversupply of ammonia may be the result of the high deamination by HAB, leading to poor ruminal N utilization efficiency and significant N losses [8]. By deaminating AA that could have stimulated the growth and therefore the ammonia utilization of the non-HAB majority [76], HAB might further reduce N utilization efficiency in the rumen.

Paster et al. [77] identified *Clostridium aminophilum*, *Cl*. *sticklandii* and *Peptostreptococcus anaerobius* as HAB, although they were already isolated earlier as strain F, strain SR [71] and strain C [78], respectively. Each of these three ‘classical’ HAB species had an abundance of 10^7^ cells/mL rumen fluid [41] or, by applying 16S rRNA hybridization, made up approximately 1% of total bacteria in the rumen [79]. However, other studies found these HAB to enumerate 5.2% and even 11.6% of total bacterial counts in cows and sheep, respectively [80]. Besides, Rychlik et al. [81] predicted HAB populations to be 4-fold higher in cattle fed hay compared to grain-fed cattle. Aside from their ionophore sensitivity, the ability to use AA as sole carbon, N and energy source is characteristic for HAB. They are unable to hydrolyze proteins and thus rely on peptide- and AA-releasing microorganisms [80]. According to Wallace et al. [82], *Eu*. *pyruvativorans* can be classified as HAB, although it grew poorly on free AA. In fact, *Eu*. *pyruvativorans* seems to prefer pyruvate as an energy source, because pyruvate is extracellularly rare in the rumen, AA utilization may be the fermentative niche of this bacterium [82].

So far, it was assumed that HAB were unable to ferment carbohydrates [80], but recent findings provide information on another type of HAB. In the rumen of steers, Bento et al. [83] observed bacterial isolates with ammonia production as high as ‘classical’ HAB [77], but with the ability to ferment carbohydrates, consequently disproving the assumption that carbohydrate-fermenting bacteria would only produce low amounts of ammonia [70, 81]. These ‘new’ HAB isolates [83] might not have been recognized, as HAB were often isolated on selective media in the past, with AA being the sole carbon source [78]. However, this does not mean that they all can only ferment AA, but have a wider metabolic role. Although phylogenetically distinct from the ‘classical’ rumen HAB [77], HAB with carbohydrate-fermenting activity are also present in swine manure and showed significantly less ammonia production when incubated with glucose, thus probably demonstrating a shift in biochemical pathways [84]. It may be possible that ruminal HAB isolates with carbohydrate-fermenting activity [83] can also shift between carbohydrate and AA utilization and would therefore have an advantage at energy generation compared to ‘classical’ HAB [77].

The ‘new’ HAB [83] were mainly assigned to *Clostridiales,* and isolates were closely related to *Cl*. *bifermentans*, *Cl*. *argentinense* or even ‘classical’ HAB [77, 83]. Therefore, the genus *Clostridium* seems to harbor more HAB species that remained unknown to date. Other studies indicate the presence of HAB in the genera *Fusobacterium*, *Eubacterium* [80], *Acidaminococcus* and *Desulfomonas* [85]. To the best of our knowledge, no reports exist on the identification of HAB species in these genera. Research on the isolation and comprehensive metabolic characterization of further HAB is required, in particular studies confirming the existence of carbohydrate-fermenting HAB in the rumen.

### Ureolytic bacteria

Urea constitutes a NC that is rapidly degraded by ruminal microorganisms and thus increases the ammonia pool in the rumen [86]. Depending on dietary composition, urea can constitute a part of the diet and therefore enters the rumen by feed intake [6, 15]. Typically, however, urea originates from the rumino-hepatic circulation and is brought into the rumen via saliva or diffusion through the rumen wall [87, 88]. This process may be useful under N-limiting conditions [87, 89], but when ruminants receive diets moderate or high in crude protein, the ammonia originating from ureolysis mainly returns to the blood rather than mixing with the rumen fluid and will be excreted into the environment [90].

Using cultivation-based techniques, several bacterial genera and species exerting ureolytic activity have been identified in the rumen, e.g. different strains of *Staphylococcus* sp., *Lactobacillus casei* var. *casei*, *Klebsiella aerogenes* and *Strep*. *faecium* with urease activities being either intracellular or linked to cell surface. Among these species, *Strep*. *faecium* showed the highest urease activity and was the most abundant of the isolated species [88]. *Howardella ureilytica* hydrolyzing urea for ATP generation was isolated from ovine rumen fluid [91] and strains of *Staph. saprophyticus* and *Micrococcus varians* were also isolated from sheep rumen and showed ureolytic activity during incubation with different NC. Besides, the majority of *Staph*. *saprophyticus* strains hydrolyzed casein and deaminated several AA [89], probably contributing to the overall proteolytic and deaminating activity in the rumen. With an abundance of at least 2 × 10^7^/mL, a ureolytic strain of *Sel*. *ruminantium* was isolated from bovine rumen [15]. Despite all these observations, the majority of ureolytic microbes are yet to be identified. Investigating the diversity of urease gene ureC in rumen fluid, digesta and papilla by NGS revealed that on average 55% of total sequences could not be assigned to any phylum. Most of these unknown sequences were found in the rumen papilla samples, where the ureolytic bacterial profile also clearly differed from those in digesta and fluid [92].

Research that considers all sampling sites in the rumen applying culture-independent methods like NGS are demanded as only limited knowledge on ureolytic rumen bacteria exists to date. However, cultivation will still be needed to definitely confirm ureolytic properties predicted with nucleic acid-based approaches [93]. Interestingly, this might be problematic as despite previous isolates being facultative anaerobes and their growth being virtually unaffected by oxygen, a considerable part of them lost the ability to undergo ureolysis during aerobic subculturing [88, 89].

## Protozoa

In addition to bacteria, protozoa represent an important part of NC-degrading microorganisms in the rumen [94]. As described by Hungate [3], protozoa are eukaryotes and mainly present in the fluid phase, though they are chemoattracted to released nutrients [95] and thus some species are transiently attached to feed particles. Rumen protozoa enumerate approximately 10^6^ protozoal cells/mL rumen fluid [96]. Estimates of protozoal abundances using microscopy can cause misidentification coupled with a low sensitivity [97]. Thus, quantification by molecular techniques targeting the 18S rDNA may be better suited, but variation in 18S rDNA copies between protozoal genera or different growth conditions can interfere [19]. Also, smaller protozoa were under-represented by 18S rDNA copies when comparing NGS data to protozoal counts [98], which demonstrates that NGS is superior for community structure analysis, but not precise quantification. In this context, an even more severe and general problem may be the procedure for obtaining protozoa samples. It was earlier shown that especially large protozoa are more retained by feed particles when rumen fluid is filtered through cheesecloth [99], which consequently leads to an overestimation of their abundance. Likewise, sedimentation funnels might not only concentrate protozoa, but also cause bias when some species are attracted to the funnel glass or do not sediment well. Thus, hereinafter mentioned abundances should be regarded with some reservations and may better serve as indications.

Protozoa are regarded as detrimental for ruminal N metabolism as they predate bacteria [96] or fungi [100] and large protozoa also engulf smaller protozoa [101], which altogether reduces ruminal N utilization efficiency. Protozoa also degrade feed protein, particularly insoluble particles [102] and thereby significantly contribute to ruminal dietary protein degradation [94]. Prins et al. [18] emphasized their role in ruminal protein breakdown as almost 20% of the proteolytic activity in rumen fluid is derived from protozoa. In contrast to NC-degrading bacteria, on a species level, little is known about protozoa or their N metabolism, which demands for investigating these microbes in detail. The highest proteolytic activity was found in vitro for *Entodinium caudatum* and *En. simplex* [103]*,* with inter-species differences in the expressed protease profiles [94]. Of all rumen protozoa, cellulolytic protozoa, in turn, exerted the lowest proteolytic activity [103]. According to protozoal fractionation and NGS, the genus *Entodinium* is predominant in cows with *En*. *caudatum* and *En*. *simplex,* both accounting for 0.5% of protozoal cells [24, 96].

Also *Dasytricha ruminantium* is highly proteolytic [94] and accounted for up to 34% of protozoal cells obtained from sedimentation funnels. It thus may have a great influence on both dietary protein degradation and the turnover of bacteria [96]. Furthermore, *Polyplastron multivesiculatum* exerted moderate proteolytic activity [94] and can account for 10–20% of the total protozoal population [102]. Members of *Isotricha* as well as *Ophryoscolex caudatus* showed less proteolytic activity, whereas that of *Epidinium caudatum ecaudatum* was higher [94, 102].

Protozoa also degrade dipeptides and *Entodinium* species exerted high dipeptidase activity, followed by *D*. *ruminantium* and members of *Isotricha* [104]. Therefore, protozoa also play a relevant role in the final stages of peptide catabolism. In a more recent study, isotrichids and entodiniomorphids showed chemotaxis towards bacterial, protozoal and soy peptides [105], which thereby indicated protozoal contribution in the ruminal breakdown of polypeptides [106].

Very little is known about deamination by ruminal protozoa [106]. Species of *Entodinium* showed deamination activity, although the quantity is negligible [107]. Hino et al. [16] observed deamination in ruminal protozoa mainly consisting of entodiniomorphids, and Forsberg et al. [108] also described low deaminase activity in protozoa sampled from rumen-cannulated cows. Concerning ureolysis, no urease activity was detected in protozoa [88, 107].

When concentrating on their contribution to ruminal N metabolism, protozoa have an adverse effect and reduce ruminal N utilization efficiency. However, parts of the protozoal population are also important for fiber degradation [109]; due to their ability to temporally incorporate starch granules, which are consequently not metabolized to organic acids, they prevent extensive pH drops and thus support a stable fermentation [110]. Finally, in consideration of a representative sampling, there is an urge to obtain deeper knowledge on the NC-degrading activity of ruminal protozoa on a species level to be able to diminish detrimental effects on ruminal N turnover.

## Fungi

Fungi constitute about 10% of microbial biomass in the rumen [111] and are crucial fiber degraders [112], especially when forages with poor quality are fed to ruminants [100, 112]. Generally, there is only marginal information on metabolic activities of rumen fungi [113] and consequently also on their contribution to NC degradation.

The fungus *Neocallimastix frontalis* PNK2, which was isolated from sheep rumen, showed high extracellular proteolytic activity, probably necessary for degrading structural proteins and for sufficient fiber degradation. Moreover, it was considered possible that proteases could modify activation of other fungus-derived CAZymes [114]. In vitro, proteolytic activity of solid rumen ingesta was considerably increased in the presence of this fungal strain [115]. Thus, the authors assumed proteolytic fungi to play an important role in ruminal protein degradation [114, 115]. Also, the ruminal fungi *Ncm. patriciarum*, *Orpinomyces joyonii* and *Piromyces communis* showed proteolytic activities during incubation with different cereal grains. Thereby, protease activity was mainly cell-associated in *Ncm*. *patriciarum* and *Piromyces communis*, whereas it was predominantly extracellular in *Orpinomyces joyonii* [116]. Likewise, Paul et al. [112] observed increased ruminal protease activities in buffaloes supplemented with *Piromyces* FNG5, although it remains unclear whether the increase originated from supplemented fungus or indigenous microbes.

No significant contribution of main ruminal fungi to in situ degradation of soybean cake and meat meal or improvements in the proteolytic activity of rumen fluid through fungi were observed by Bonnemoy et al. [117]. Likewise, Michel et al. [118] stated that rumen fungi have limited abilities to degrade proteins; however, several fungal isolates exerted endo- and exopeptidase activities [118] and may therefore promote ruminal peptidolysis. Additionally, a higher fungal diversity during the increment of protein supply to dairy cows [30] might indicate that ruminal fungi partly benefit from high protein provision.

Besides its metabolic activity, knowledge on the abundance of a microorganism can help to evaluate its contribution and meaning to rumen metabolism. Because previous studies only enumerated total abundances [30], studies quantifying ruminal fungi on a species level or with regard to their metabolic activity are needed. In the past, quantification of rumen fungi via microscopy may have led to erroneous results, as flagellate protozoa can be identified as fungal zoospores [100]. Thus, DNA-based methods targeting the 18S rDNA or internal transcribed spacer 1 [97] represent attractive alternatives for quantification and diversity analysis. Subsequently, fungal DNA amount or copy numbers of internal transcribed spacer 1 can be converted to estimate the fungal biomass in the rumen [119, 120].

## Interactions among nitrogenous compound-degrading rumen microorganisms

It is well established that ruminal microorganisms are continuously interacting, but so far these interactions are not fully understood [30]. In the following, relevant interactions between or with the contribution of NC-degrading rumen microorganisms will be reviewed briefly. Basic interactions like interspecies H_2_ transfer [111] will not be included.

Wallace [42] early showed the existence of reciprocal interactions between NC-degrading rumen microbes, as different proteolytic bacteria grew better in combination than alone, which was ascribed to an increased cooperative hydrolysis or nutritional interdependences. Hyper-ammonia producing bacteria, both ‘classical’ [77] and ‘new’ [83] HAB, depend on proteolytic species that supply AA to them [80, 83] as, except one isolate [121], they are unable to hydrolyze proteins to sustain growth. Moreover, *Cl*. *aminophilum* and *Psc*. *anaerobius* produced much higher amounts of ammonia when grown with peptidolytic *P*. *ruminicola* or *P*. *bryantii* [122], thus indicating the dependence on peptidolytics.

Interactions between NC-degrading microorganisms are not always beneficial, but can impair other species, e.g. by bacteriocins [123]. For instance, bovicin HC5, a bacteriocin formed by *Strep. bovis* HC5 catalyzing the potassium efflux from cells, inhibited growth and ammonia release of *Cl. aminophilum* in pure cultures [124], but also decreased ammonia production of mixed ruminal bacteria in vitro [123]. Strains of *B*. *fibrisolvens* produced a variety of bacteriocins including JL5, which suppressed *Cl*. *sticklandii* as well as cellulolytic *Ruminococcus albus* and *Ruminococcus flavefaciens* [125], which might be explained by the competition for carbohydrates. Generally, the formation of as well as sensitivity against bacteriocins seems to be strain-specific [123, 125].

Although excessive deamination is regarded as detrimental for efficient N utilization [126], there is also a need for deaminating bacteria and linkages between cellulolytic and deaminating rumen microbes. As ammonia represents the sole N source for cellulolytic bacteria [7], deamination is an important process to provide this NC. Branched-chain volatile fatty acids (BCVFA), which are formed during deamination [127], have stimulatory effects on predominant cellulolytic microorganisms and are crucial for their growth [128]. Then again, these deamination processes remove AA that could have promoted growth of cellulolytics [76]. Bacteria like *Strep*. *bovis* exert proteolytic activities to gain access to starch granules surrounded by protein matrices [38]. Thus, this also affects nutrient provision of other amylolytic microorganisms, which are not capable of degrading such matrices.

On the other hand, proteolytic bacteria can benefit from fiber degraders, as maximal cell wall protein degradation was observed when proteolytic and cellulolytic bacteria were incubated together. It was hypothesized that potentially degradable proteins are protected by structural polysaccharides and become available for proteolytic microorganisms through cellulose degradation [129]. Thereby, rumen fungi may also play a role, as they degrade cell wall structures [100], and can therefore provide access to actually surrounded proteins.

Protozoa predate bacteria [96] and fungi [100], but by degrading insoluble dietary proteins, protozoa promote the growth of peptidolytic and deaminating bacteria, which utilize peptides and AA from protozoal proteolysis. Thereby, protozoa may enhance the deaminating activity of HAB [9] and thus reduce the efficiency of N utilization in the rumen by two modes of action: the predation of bacteria and fungi as well as the release of AA into the rumen. Similar patterns of interaction may occur between peptidolytic or deaminating bacteria and proteolytic fungi that release NC from their protein breakdown [114]. Dehority et al. [130] stated a general negative interaction between fungi and bacteria, as both form inhibitory substances to limit the growth of the other. However, the administration of *Piromyces* FNG5 caused a 2.5-fold increase of bacteria in the rumen of buffaloes [112]. Therefore, the existence, type and extent of interaction between fungi and bacteria may be specific for species or even strains [131] and must be evaluated individually.

## Manipulating factors on nitrogenous compound-degrading rumen microorganisms

Several factors influence the rumen microbiota within a ruminant: Age [132], geographical localization, host species [24], breed [133] and diet [24]. Thereby, diet has the strongest influence [24] and is thus of great importance in livestock production.

The meaning of the rumen microbiota with regard to high performance in livestock production was highlighted by the observation that ruminal bacteria communities of steers with higher feed efficiency (defined as “the difference between an animal’s actual feed intake and its expected feed requirements for maintenance and growth over a specific test period” [134]) were more similar between individuals and clearly separated from ruminal bacteria communities of inefficient animals [133]. Therefore, a specific rumen microbiota composition may be significant for satisfying performance of animals. Another study [135] indicated the potential of rumen microbes to influence quality characteristics of milk. The scientists observed high correlations between milkfat yield and the *Firmicutes* to *Bacteroidetes* ratio, which were still present at the genus level [135]. Both studies emphasize the influence of rumen microorganisms on the host’s physiology and thus the meaning of shaping the microbial composition and its activity to improve nutrient and energy use.

Concerning ruminal N utilization, manipulating the number and activity of NC-degrading microorganisms is of particular importance, especially as ammonia release in the rumen often exceeds its efficient utilization [126] and consequently results in high N losses and a waste of resources. Attwood et al. [27] stated the importance of altering the abundance of NC-degrading microorganisms as microbial enzymes would be expressed permanently and thus controlling ruminal NC degradation by affecting enzyme expression could not represent a promising strategy [26]. Bladen et al. [70], in turn, assumed that increased ammonia production was caused by higher deaminase synthesis without alterations in microbial abundances; therefore, influencing enzyme expression would be the better opportunity to control intra-ruminal N recycling. So far, different approaches have been applied to improve ruminal N utilization using one of these two strategies and will be discussed in the following.

## Diet composition

### Selection of forage species

When preparing ruminant diets, selection of forage species affects the composition of NC supplied to the rumen [136] and thereby also the ruminal microbiome and efficiency of N utilization [127]. In the case of legumes, red clover (*Trifolium pratense*) expresses polyphenol-oxidase (PPO), an enzyme that causes the formation of protein-phenol complexes when plant tissues are damaged [137]. This increases the proportion of ruminally undegraded dietary crude protein (RUP) [138], which is still digestible in the small intestine and thus an available N source for the host. Moreover, red clover phenolic extract inhibited the growth of *C. sticklandii* cultures in vitro [139], which along with PPO, may increase N retention in ruminants. The effect of PPO on ruminal proteolysis was demonstrated in vitro, as the inclusion of red clover to timothy grass-based diets lowered the ammonia to insoluble-N ratio, indicating a limited protein degradation [140]. Likewise, the production of ammonia and i-valerate was lower in fermenters supplied with cocksfoot (*Dactylis glomerata*), a grass species high in PPO, when compared to fermenters incubated with the low PPO grass species tall fescue (*Festuca arundinacea*) [141]. Cocksfoot may hence be an important grass species on permanent pasture used for both grazing and silage production. Likewise, red clover may be an attractive legume for arable pasture areas to improve the RUP supply from forage plants.

### Synchronization of dietary energy and nitrogen

Combining different feedstuffs to synchronize the provision of dietary energy and N [142] may also affect certain groups of NC-degrading microorganisms. In diets containing highly degradable NC and less rapidly available energy, e.g. water-soluble carbohydrates (WSC), HAB might increase in activity and abundance [143] as they probably benefit from their ability to utilize AA energetically without being dependent on carbohydrates [71, 78, 80, 83].

The effects of synchronization are not consistent in literature [142, 144], but increased microbial N flow to the duodenum and reduced ruminal ammonia concentration have been reported for the combination of legume silage and grass silage high in WSC [141], thus suggesting less deamination [8] and the direct incorporation of AA into microbial protein [7]. Likewise, microbial N flow at the duodenum tended to increase with a more synchronous supply of dietary energy and N to steers, although ruminal ammonia concentration was not reduced [145]. Studies focusing on whether there are also adaptions in the rumen microbiome are lacking, but could reveal possible linkages that help to reduce N losses from the rumen.

The synchronization of dietary energy and N could reduce detrimental consequences of high deamination activity from HAB as it supplies sufficient energy for microbial utilization of ammonia. Secondly, adequate provision of dietary energy may nullify the advantage of HAB to generate energy from AA. This may reduce their abundance along with long-term alterations of the ruminal microbiota composition and could also explain the predicted 4-fold lower HAB population in cattle fed high-grain diets compared to hay-fed cattle [81]. Moreover, one can speculate whether HAB capable to ferment carbohydrates [83] shift from AA to carbohydrates as the preferred substrate for energy generation and thus still be present in the rumen but exerting a different metabolic pathway with less deamination.

### Application of fats

Diets containing higher proportions of fats with unsaturated fatty acids, commonly termed oils, are already used to increase the energy supply to ruminants [146]. Although this does not help to cover the energy needs of rumen microbes [147], fat supplementation may influence ruminal N metabolism [146]. For instance, the addition of 26 mL/d of linseed oil to a basal diet for sheep almost eliminated all rumen protozoa and improved the efficiency of bacterial protein synthesis by more than 50%, whereas increasing linseed oil supplementation to 40 mL/d showed no effect on the efficiency of bacterial protein synthesis [148]. Other studies even reported the increased abundance of proteolytic bacteria and the increased formation of ammonia when feeding linseed or soybean oil to dairy cows [149]. Therefore, the effects of fat supplementation on NC-degrading microorganisms seem to be variable and difficult to predict. Besides, detrimental impacts like reduced ruminal degradation of organic matter [148] or hemicelluloses [147] and subsequently less availability of dietary energy in the rumen, were repeatedly documented for oil supplementation. This can be explained by the general toxicity of unsaturated fatty acids on rumen microorganisms [147] and might outweigh the positive impact on intra-ruminal N recycling.

## Feed treatment

### Wilting

Wilting forages is an efficient way to reduce energy losses during ensiling [150], but also influences the composition of NC in silages [151, 152]. Both may affect ruminal NC-degrading microorganisms via variation in the energy supply [152] and differing percentages of true protein in silages [151], thereby influencing the quality of N supply to the rumen. Fast wilted silages have higher true protein contents and lower ruminal ammonia concentrations during in vitro and higher RUP values during in situ incubation [151]. Likewise, grass silages with high contents of free AA (> 300 mmol/kg dry matter) and less true protein resulted in higher concentrations of ammonia and BCVFA along with a lower efficiency of N assimilation into microbial protein by liquid-associated bacteria in vitro [143].

In grass silages, wilting also increased WSC [150, 152], which can enhance microbial protein synthesis in the rumen by a higher provision of dietary energy [153]. Interestingly, wilting is assumed to promote PPO activity in red clover [137], thereby increasing RUP by forming phenol-bound proteins [138]. However, no enhanced PPO activity was obtained when wilting cocksfoot [154] suggesting that effects on PPO activity depend on plant species. To the best of the authors’ knowledge, there is a lack of research on the effect of differently wilted forages on the ruminal microbiota composition. So far, only microbial metabolite productions have been investigated, but comprehensive experiments analyzing both microbial abundances and metabolites are indispensable to optimize forage conservation in the future.

### Organic acid treatment

Influencing the ruminal microbiome by processing feedstuffs with organic acids was found to be an effective option during recent years [22]. Acids alter the solubility and protein structure, thereby affecting the quantity and quality of N supplied to the rumen [155]. Barley treated with lactic acid reduced BCVFA concentration in vitro [22, 68], but did not affect ammonia concentration [22]. Deckardt et al. [22] concluded a decreased AA catabolism, although addition of lactic acid did not affect the abundance of *M*. *elsdenii* or other NC-degrading microorganisms like *Prevotella* or *Entodinium*. Besides, fiber degradation was enhanced in acid-treated barley [22], which emphasizes the benefit of processing concentrate with organic acids. Concerning the degradation of soybean meal protein, treatment with 5% propionate reduced proteolysis as well as numerical concentrations of ammonia and i-valerate in vitro [155], indicating reduced metabolic activities of proteolytic and deaminating microorganisms [127, 143]. Manipulating the structure and solubility of proteins through acid treatment seems to be a feasible approach to alter the cascade of NC breakdown in the rumen. Together with the observations for fiber degradation, findings so far give evidence for an improved N retention, which should be pursued in further studies addressing total N flows.

### Heat treatment

Heat treatment constitutes a further option to affect ruminal N metabolism. Treating barley grain with 55 °C for 48 h decreased protein degradation, the concentration of i-valerate as well as the abundance of *Prevotella* and total protozoa in vitro. However, the degradation of organic matter was also lowered [22]. Extensive heat treatments should be applied carefully, as exposing rapeseed meal to 130 °C or 140 °C for five minutes led to high RUP contents, but the 140 °C treatment also caused poor intestinal protein digestibility [156]. Thus, N becomes unavailable for the rumen microbiota and the host, leading to unnecessary N losses and environmental pollution. Duration and intensity are decisive for the effect of heat treatments on NC and thereby determine whether they improve N utilization in ruminants or actually cause the opposite result.

## Feed additives

### Plant bioactive lipid compounds

Plant bioactive lipid compounds (PBLC), commonly but misleadingly termed ‘essential oils’ [157], are secondary plant metabolites that are not necessary for plant growth and characterized by a vast diversity [158]. So far, a variety of PBLC has been shown to reduce ruminal methane production [159], but also to affect NC-degrading microorganisms by microbicidal or microbiostatic effects [8].

Reduced deamination as well as concentrations of ammonia [8, 159, 160] and BCVFA were observed with PBLC supplementation in vitro [8, 159]. Application would hence mean an effective dietary strategy to prevent inefficient N utilization by ruminants. However, PBLC do not necessarily decrease ruminal ammonia concentrations or deamination [8, 159, 161], and can even have opposite effects [161].

Plant bioactive lipid compounds cause substantial alterations in the ruminal microbiota, but it is not clear whether they are suited to shape the NC-degrading microbial population in the rumen. *Streptococcus bovis* is relatively resistant against a multitude of PBLC including thymol [162], clove oil, origanium oil [8] as well as a commercial blend of PBLC [160]. *Ruminobacter amylophilus*, *B. fibrisolvens* and *Sel. ruminantium,* as well as *P. bryantii* and *P. ruminicola,* were highly sensitive against clove and origanium oil [8]. Likewise, protozoa showed high sensitivities against these two substances [8, 159] and also against eucalyptus, garlic and peppermint oil in vitro [159]. Consequently, ruminal peptidolysis and proteolysis may be added to the statement of Calsamiglia et al. [158] that the majority of PBLC could affect ruminal deamination.

Abundances of deaminating bacteria, particularly HAB, were often reduced by PBLC supplementation and may explain reduced ammonia concentrations [160]. Origanium oil decreased 16S rDNA copies of *M. elsdenii*, *Cl. aminophilum* and *Cl. sticklandii* in a bovine rumen fluid-based in vitro system [8] and the PBLC blend of McIntosh et al. [160] inhibited the growth of *Cl. sticklandii* and *Psc. anaerobius* pure cultures. However, in contrast, *C. aminophilum* was not affected by different PBLC blends [160, 163].

The aforementioned results were all obtained during in vitro experiments and thus must be evaluated under in vivo conditions. Thereby, the supplementation form needs to be considered as well, as it can influence the effect on ruminal microorganisms. For instance, 16S rDNA copies of *Prevotella* spp. and *Cl*. *aminophilum* declined in sheep fed pelleted rosemary leaves (*Rosmarinus officinalis* L.), whereas 16S rDNA copies of the same species were not affected when sheep received the same dosage as pure ‘rosemary essential oil’. The authors suggest that differences in chemical composition between the supplementation forms are responsible for the deviating effects [164]. Also, ‘rosemary essential oil’ might not have emulsified properly with the rumen fluid and lacked effectiveness as it floated on top.

Generally, the effects on ruminal NC-degrading microorganisms seem to be specific for the applied PBLC. However, contradictory findings between studies investigating the same PBLC [159, 161, 163] clearly illustrate that the underlying modes of action are poorly understood and a definite statement in this regard is impossible. Consequently, as claimed previously [8], there is an urgent need for systematic studies on PBLC using standardized conditions to obtain reliable knowledge on the effects on ruminal N metabolism and microorganisms. Hereby, PBLC should also be critically evaluated for their effect on feed digestion, as several PBLC combinations reduced dry matter digestibility in vitro [163].

### Condensed tannins

As Patra et al. [165] have summarized, supplementing ruminant diets with tannins can influence ruminal metabolism and consequently also intra-ruminal N recycling. In fact, condensed tannins (CT) have protein binding effects at pH 3.5–7.0 [166] leading to reduced proportions of soluble protein in the rumen and probably increased RUP values [106]. In the abomasum and the proximal duodenum, dietary protein would be available for the host due to low pH values causing the breakdown of these complexes. However, the risk of repeated formation of protein-tannin-complexes or tannin-binding to the host’s enzymes in lower gut sections remains present [166].

In pure cultures, bacteria were unable to degrade proteins when incubated with calliandra CT (*Calliandra calothyrsus*) [121], and a protein-preserving effect of sainfoin CT on ruminal proteolysis was also evident using ovine rumen fluid, as the ammonia to insoluble-N ratio was lowered [140]. Likewise, quebracho CT reduced ammonia concentration in vitro [167]. All these observations may be explained by two factors: the formation of protein-tannin-complexes [121, 166] and morphological alterations of bacterial cell walls [168] suppressing the growth and proteolytic activity of NC-degrading bacteria [121, 168, 169].

On the other hand, as ammonia constitutes the main N source for cellulolytic bacteria, an excessive tannin-induced protein protection bears the risk of ruminal ammonia concentrations below the critical level for sufficient forage digestion [170] and would have adverse effects on the host’s supply with dietary energy and nutrients. Besides, CT can directly inhibit ruminal cellulolytic species [170], their cellulase activity [171] or form complexes with lignocellulose [170].

Feeding CT-rich plants often reduces feed intake [31] due to the reduced palatability and lower degradation rates [172]. Thus, by directly feeding CT-rich plants, it may be difficult to achieve tannin concentrations causing adequate protection of dietary protein in the rumen; however, supplementing purified CT extracts to ruminants should be an option to affect ruminal degradation of NC, particularly of proteins. These positive effects must then be weighed carefully against the potential detrimental impact on cellulolytic rumen species.

### Saponins

Besides PBLC and tannins, saponins are a third group of secondary plant metabolites with bioactive functions [158]. Several saponins were evaluated as feed additives in animal nutrition [173–175], but as with PBLC, the results are not consistent. Quillaja saponins (*Quillaja saponaria* Molina) lowered ammonia concentration by 20% in vitro, which was probably due to a reduction of protozoal 18S rDNA copies [8]. It is assumed that saponins form complexes with sterols in the protozoal membrane surface, which thereby becomes disrupted [176] and leads to cell death. Similarly, *Yucca schidigera* saponins inhibited protozoal predation and reduced ammonia concentrations in vitro [177]. In contrast to the in vitro data [177], protozoal counts in rumen fluid were not altered, when *Yucca schidigera* saponin extract was supplemented to dairy cows [173]. Tea saponins, however, reduced ruminal ammonia concentration and protozoal 18S rDNA copies when fed to sheep [174].

The effects of saponins on several NC-degrading bacteria are even less clear. Quillaja saponins increased the 16S rDNA copies of *Rb*. *amylophilus*, *Sel*. *ruminantium*, *P. ruminicola* and *P*. *bryantii* [8], what was also observed for *Prevotella* [175] and *P. bryantii* [177] during in vitro application of *Yucca schidigera* saponins. In contrast to quillaja saponins [8], *Yucca schidigera* saponins suppressed the growth of pure cultures of *Rb. amylophilus* [178] and also that of *B. fibrisolvens* [177]. 16S rDNA copies of *M*. *elsdenii* and ‘classical’ HAB [77] were not affected by quillaja saponins, which seems contradictory in view of the reduced ammonia concentration [8]. The authors suggested the decrease of protozoa to be responsible. However, reductions of proteolytic and deaminating bacteria that were not targeted by qPCR, e.g. ‘new’ HAB [83], could also be causative [8]. Additionally, one can speculate whether saponins have suppressing effects on metabolic activities but not abundances of NC-degrading bacteria.

So far, the use of saponins may be an option to modulate ruminal NC degradation in a beneficial way, but inhibitory effects on protozoa or bacteria seem to depend on dosage as well as saponin type and remain poorly understood [8]. Under practical conditions, however, the ability of saponins to reduce feed intake [173] clearly limits their application to dairy diets.

### Anacardic acids

As summarized by Kobayashi et al. [179], anacardic acids predominantly present in the by-products of cashew (*Anacardium occidentale*) and ginkgo (*Ginkgo biloba*) nut production, are discussed as modifiers of rumen fermentation. Anacardic acids are characterized as a group of few closely related organic compounds, differing in saturation and side chain length [180]. A first in vitro study investigating the effect of anacardic acid containing ginkgo by-products found decreased concentrations of ammonia, which may be caused by high sensitivities of ‘classical’ HAB [77] against anacardic acids [181]. However, the effects on other ruminal NC-degrading microbes are less clear and even contradictory in parts. For example, 16S rDNA copies of *M*. *elsdenii* and *Sel*. *ruminantium* increased in vitro when fermenters were supplied with either ginkgo extract [181] or cashew nut shell liquid [182]. 16S rDNA copies of *P. ruminicola* increased with ginkgo extract [181], but declined when cashew nut shell liquid was added [182]. This inconsistency may be explained by differences in the structure of anacardic acids contained in ginkgo and cashew by-products [181] or also by the presence of other antimicrobial compounds [179]. As both studies used similar diets for the in vitro incubation as well as bead beating-based DNA extractions and identical primers for qPCR analysis, laboratory procedures may not have been a contributing factor for the deviating microbial abundances. Therefore, further studies are indispensable to evaluate whether anacardic acids are an option to shape the ruminal NC-degrading microbiota and which structural form is most effective.

### Bitter substances

Although bitter substances were early found to have antimicrobial properties [183], their consideration as modulators of the rumen microbiota is new and scarcely explored. First in vitro investigations by Flythe [184] observed hops flowers (*Humulus lupulus* L.) and hops extract to inhibit ammonia production in mixed rumen fluid, as well as growth and ammonia production in pure cultures of ‘classical’ HAB [77]. These suppressing effects are likely caused by humulone and lupulone, which are the main bitter substances in hops, and are also known as α-acid and β-acid, respectively [184]. Additionally, growth of *Strep. bovis* was inhibited by lupulone when cultivated in pure culture [185]. Likewise, ammonia production in rumen fluid incubated with spent craft brewer’s yeast was lower than that with baker’s yeast. This supports the assumption that hops bitter substances can decrease ruminal ammonia production and indicate reduced deamination [186].

The target site of humulone and lupulone is the cell membrane’s lipophilic region, where they cause membrane leakage and consequently cell death [183]. However, except for ‘classical’ HAB [77] and *Strep. bovis*, no information is available regarding their effect on other rumen microorganisms. As the supplementation with two hops cultivars not only decreased in vitro degradability of crude protein by up to 36%, but also degradability of dry matter by up to 33% [187], inhibiting effects on other parts of the rumen microbiome are likely. Thus, bitter substances like humulone and lupulone could provide an opportunity to affect ruminal N metabolism in the future; however, sparse knowledge bases on in vitro trials and hitherto an assessment cannot be made.

### Ionophores

Since their ban as feed additives in the EU in 2006 [188], ionophores only represent an option in other parts of the world, e.g. North America where ionophores, predominantly monensin, are widely applied in beef production [189]. Ionophores are described as a heterogeneous group of membrane-active molecules impairing transmembrane concentration gradients [190]. Concerning their effect on protozoa, it must be distinguished between short-term and long-term effects. Although naïve protozoal populations were nearly completely eliminated in vitro [16], repeated application of monensin did not have an effect on protozoal cultures [191]. These adaption patterns do also apply for in vivo long-term monensin application [192]. Thus, also decreased ammonia concentrations observed shortly after a monensin-induced protozoa reduction [16] may not last and return to pre-treatment level. This might even be the case if protozoa would stay absent as bacteria proliferating in the absence of protozoa could replace protozoal activity [104].

Although there is no clear-cut difference in the susceptibility against monensin between gram-negative and gram-positive bacteria [193], gram-negative bacteria are generally more resistant [192, 194] due to their outer membrane structure and cell wall constitution [194]. However, also cell flocculation or synthesis of protective extracellular polysaccharides affect the effectiveness of monensin and can occur in both gram-positive and gram-negative bacteria [193]. Thus, future studies should address microbial taxa [195] to provide a superior picture about the monensin susceptibility of NC-degrading microorganisms in the rumen.

The gram-positive ‘classical’ and ‘new’ HAB [77, 83] are monensin-sensitive [71, 78, 83] and monensin is undoubtedly effective at reducing deamination [196], ammonia concentration and abundances of ‘classical’ HAB [77] in vitro [16, 160, 196] and in vivo [79, 197].

In vitro application of the ionophore hainamycin decreased 16S rDNA copies of *B. fibrisolvens*, *Cl*. *sticklandii*, *Cl*. *aminophilum* and *Psc*. *anaerobius*, whereas *M*. *elsdenii* was unaffected and *P. ruminicola* increased [198]. Actually, these shifts can be considered beneficial as they were accompanied by decreased ammonia concentration, deaminase activity and proportions of BCVFA in total volatile fatty acids.

Altogether, the aforementioned findings suggest a general ability of ionophores to influence NC-degrading bacteria and underline their potential to reduce ruminal N wastage. Nonetheless, research on long-term application of ionophores should confirm lasting alterations in abundance and activity of NC-degrading rumen microbes.

### Probiotics

Probiotics are defined as live microorganisms that confer a health benefit on the host by improving its intestinal microbial balance [199] and are widely used in animal nutrition [200]. Applying qPCR, 16S rDNA copies of *Rb. amylophilus* and *Strep*. *bovis* decreased in steers supplied daily with viable *Saccharomyces cerevisiae* I-1077 through rumen cannulas. 18S or 16S rDNA copies of total protozoa and *Sel. ruminantium*, in turn, increased due to the supplementation [201]. Concerning N metabolism, proteolytic and peptidolytic activities of *P*. *albensis*, *B*. *fibrisolvens* and *Strep*. *bovis* decreased during in vitro incubation with viable *S. cerevisiae* I-1077 [202]. However, despite various alterations on a microbiological level, other studies reported no improvements of the amount and AA composition of microbial N reaching the duodenum in dairy cows supplemented with live yeast culture of *S. cerevisiae* [203]. Therefore, comprehensive studies evaluating the effects of *S. cerevisiae* on ruminal NC-degrading microorganisms are encouraged as probiotic effects seem to be strain-specific [204]. Additionally, the combination of probiotics either with prebiotic substances, leading to so-called synbiotics [200], or with plant extracts (e.g. tannins) could enhance the effects in ruminants [205].

A chance to inhibit AA catabolism through probiotics was indicated by Callaway et al. [196], who found that the *L. lactis*-derived bacteriocin nisin [206] suppressed deamination and the growth of *Cl*. *aminophilum* in vitro. Thus, supplying ruminants with bacteriocin-producing probiotics might particularly alter deamination, but studies on the identification of strains that inhibit growth [205] and activity of HAB are still pending. Because existing data [123–125, 196] were only obtained from in vitro experiments, more efforts must be made to evaluate such mechanisms in vivo. However, various interactions with other microbes and the host as well as the general high complexity of the rumen microbiome could make it hardly possible to relate any measured effect to bacteriocins.

To expand the field of probiotic candidates, the transfer of microorganisms from one ruminant species to another might constitute a further option to improve ruminal N metabolism. Administering a fungal strain from wild blue bull (*Boselaphus tragocamelus*) to buffaloes (*Bubalus bubalis*) increased N retention along with higher protease activity, but equal ammonia concentration [112], indicating a higher breakdown of dietary crude protein but concomitantly enhanced ruminal N utilization. Besides, the increase of cellulolytic and hemicellulolytic bacteria [112] may have contributed to higher N retention, as it could lead to a more synchronized fermentation of carbohydrates and NC. It is noteworthy that microbes showing effectiveness in one ruminant species can fail to colonize the rumen of other species as the establishment of exogenously added microorganisms is difficult [67].

### Metals

A variety of metal compounds is added to ruminant diets to meet mineral element requirements [207]. Besides, metal ions have antimicrobial effects and in case of NC-degrading microorganisms iron, copper, tin and chromium decreased bacterial dipeptidase activity by interacting with sulfhydryl groups or by displacing the metal ion from the enzyme [208]. Although iron effectively reduced dipeptidase activity in pure cultures of *P*. *albensis* [208], the effect may be nullified in rumen fluid as lactobacilli can take up high amounts of iron [209]. Besides the effects on rumen bacteria, Brade et al. [210] described defaunating effects of zinc-enriched diets in the rumen of dairy cows. Likewise, Mihaliková et al. [211] found high sensitivities of *En. caudatum* cultures against copper and chromium.

Lead, cadmium and mercury also inhibit microbial dipeptidase activity [208], but are irrelevant for controlling ruminal N metabolism due to the tremendous toxicity to humans and animals [212].

Metal ions are probably not specific to peptide metabolism [208] and therefore impair other ruminal microorganisms as well. Besides, some metals are also not suitable for every host; for example, several sheep breeds have low tolerance levels for copper [213]. Therefore, extensive metal utilization to improve N utilization in ruminant production is unlikely. This is particularly true as the majority of metals will be excreted via feces when fed in higher concentrations [214], thus only shifting but not mitigating the problem of environmental pollution.

## Vaccination

Vaccination against protozoa has been examined to reduce bacterial predation thereby improving the efficiency of N utilization [215]. Although applied vaccines increased specific immunoglobulins (Ig)G titers in plasma and saliva, they failed to decrease the ruminal ammonia concentration and protozoal counts in vivo. This failure might also be caused by Ig breakdown in the rumen; although IgG were resistant against degradation for eight hours in vitro [215], the in vivo situation may be different. Analogous to the secretory component of IgA, which conserves this Ig from proteolysis in the gut [216], a protective component may also improve the effect of vaccination against protozoa in the rumen. Though, it should be noted that a general absence of protozoa does not necessarily mean an improvement of ruminal metabolism as, besides their role in bacteria, fungi and NC breakdown, protozoa are also important for sufficient ruminal fiber degradation [109]. Thus, as already claimed [96, 106], it is desirable to inhibit specific protozoal species to improve N utilization and simultaneously maintain fiber degradation. Future approaches should also concentrate on long-term bacterial-protozoal interactions as bacteria may replace protozoal dipeptidase activity in the rumen [104].

A recent vaccination strategy targets the ruminal breakdown of urea and showed reduced ureolysis and ammonia concentration in vitro when incubating rumen fluid of cows that had been vaccinated against urease. Accordingly, ruminal urease activity decreased by 17% after vaccination in vivo [86] leading to the assumption that immunization against urease has the potential to control ruminal ureolysis. In this study [86], samples were taken from rumen fluid, but provided that anti-urease Ig are not only present in saliva, but also diffuse through the rumen wall, it may be interesting to investigate effects on the epimural ureolytic bacteria, which therefore should be strongly affected. Nevertheless, for evaluating the overall benefit of urea vaccination, effects on total N utilization must be analyzed, too.

## Future perspectives

This review discussed dietary factors by which ruminal NC degradation processes and related microorganisms can be influenced in adult animals. As a future perspective, manipulation of the rumen microbiome in young pre-ruminants may become an opportunity to shape ruminal metabolism and microbiota in adult animals. As a specific rumen microbiota composition is associated with improved animal performance, corresponding microbial compositions could be used as inoculum or feed additive in young ruminants to establish a favorable microbiome. Consequently, the adult ruminant may achieve an enhanced performance, not only with regard to energy utilization but also in terms of ruminal N utilization.

Inhibiting the establishment of only specific microorganisms in the evolving rumen could be a second approach for shaping the rumen microbiota in a more permanent manner. Thereby, other rumen microbes could occupy the niche of inhibited species consequently excluding them from the adult microbiota. Suppressing the colonization of HAB already in the developing rumen may become a beneficial strategy regarding intra-ruminal N recycling.

To investigate such options in the future and for further research on the rumen ecosystem in general, studies employing large sample numbers will be required to overcome the confounding effect of natural animal-to-animal variation and enhance the statistical power of rumen microbiota-related studies. However, the need for cannulated animals considerably limits the broad examination of the rumen microbiome and stomach tubing, the main source for rumen microbiome in many studies, is misleading because it under-represents particle-associated microorganisms. Recent findings indicate that non-invasive regurgitated ingesta samples are suitable for the precise prediction of rumen microbiota compositions [217]. Therefore, this can constitute an appropriate sampling method for determining rumen microbial communities on a large scale. However, phylogenetic information is unable to fully explain all underlying mechanisms or relevant activities of ruminal NC-degrading microorganisms. Thus, besides capturing the microbial composition in the rumen, the concurrent pursuit of a functional classification may be decisive in future to further improve our understanding of intra-ruminal N recycling and consequently how to manipulate it beneficially.

## Conclusions

In summary, ruminal NC degradation is not a wasteful process per se, but as ruminal ammonia concentration often exceeds the microorganisms’ capacity to incorporate, high N losses are the consequence. Controlling intra-ruminal N recycling may not only help improving N utilization and optimizing ruminant livestock production, but also contributes to a more sustainable agriculture due to less N-containing emissions and lowered resource input. Thereby, the superordinate aim is the sufficient suppression of ruminal NC degradation, particularly deamination, along with maintaining an adequate N provision for the rumen microbiota to ensure high fiber degradation and host supply with microbial protein. The information on NC-degrading rumen microbes is still very limited and investigations on their phylogenic and functional characterization are far from complete. It is known that ruminal NC-degrading microorganisms mainly belong to bacteria, but also to protozoa and fungi. Bacteria are present at all stages of ruminal NC breakdown. Because of high deamination rates, HAB particularly contribute to excessive ruminal ammonia release and subsequent poor N utilization. So far, these bacteria were assumed to show low abundance in the rumen and to use AA as energy and N sources, but not carbohydrates. However, recent findings indicate the presence of HAB able to ferment both AA and carbohydrates. Protozoa are particularly active at degrading insoluble proteins, dipeptides and are responsible for bacterial and fungal predation. The contribution of fungi to ruminal NC degradation is smaller and concentrates on proteo- and peptidolysis. Due to nutritional interdependences and competing demands, NC-degrading microorganisms are continuously interacting with each other and further members of the rumen microbiota. By using different dietary strategies or vaccination, the composition as well as the metabolic activity of NC-degrading microorganisms can be manipulated. Thereby, the targeted composition as well as the treatment of feedstuffs provide promising approaches. Besides, a variety of feed additives including probiotics, condensed tannins or PBLC may constitute effective tools for controlling ruminal N metabolism. The limited existence and partial inconsistency of results confound the exact evaluation of so far investigated ways to manipulate NC-degrading microorganisms. Beyond that, too many approaches being effective in vitro were not followed up in vivo but this should be undertaken in the future. Thus, systematic and comprehensive studies investigating the composition and metabolism of the rumen microbiome are crucial to obtain a deeper knowledge that will subsequently allow a targeted manipulation. Thereby, omics and qPCR will play a leading role, supported by new developments in sampling techniques.

## Abbreviations

AA: Amino acid
*B*.: *Butyrivibrio*
BCVFA: Branched-chain volatile fatty acids
*Cl.*: *Clostridium*
CT: Condensed tannin
*En*.: *Entodinium*
*Eu*.: *Eubacterium*
HAB: Hyper-ammonia producing bacteria
Ig: Immunoglobulin
*L*.: *Lactobacillus*
*M*.: *Megasphaera*
N: Nitrogen
NC: Nitrogenous compound
*Ncm*.: *Neocallimastix*
NGS: Next-generation sequencing
*P*.: *Prevotella*
PBLC: Plant bioactive lipid compound
PPO: Polyphenol oxidase
*Psc*.: *Peptostreptococcus*
qPCR: quantitative real-time polymerase chain reaction
*Rb*.: *Ruminobacter*
RUP: Rumen-undegraded dietary crude protein
*S*.: *Saccharomyces*
*Sel*.: *Selenomonas*
*Staph*.: *Staphylococcus*
*Strep*.: *Streptococcus*
WSC: Water-soluble carbo-hydrates
